# Lessons from the recent Journal Citation Report figures

**DOI:** 10.3109/03009734.2010.505109

**Published:** 2010-07-19

**Authors:** Arne Andersson, Gunnar Ronquist

**Figure 1.**
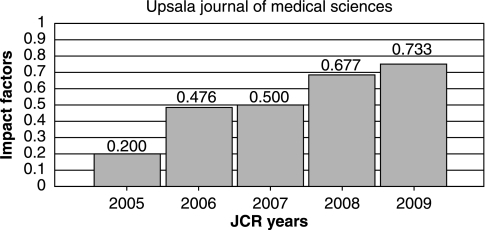
A graph imported from ISI Web of Knowledge demonstrating the impact factor trend for Upsala Journal of Medical Sciences. The previous all-time high, 0.688, was from the year 2002.

Mid-June has always been a period of great hope or perhaps fear for editors of medical journals. This is the time of the year when new figures for impact factors are released from the ISI Web of Knowledge. However, most editors these days have a good insight into the development of this very much debated instrument for evaluation of a journal's relative importance for the distribution of scientific information. By consultations of the continuous updating of citation figures for each individual article of their journals in either Web of Science (Thompson & Reuter) or SCOPUS (Elsevier) it is possible to make fairly good estimates of the figures to come. It was therefore not a great surprise to us when the new impact factor figure this time had increased to a record high 0.733. This also means that we have now increased our impact figure for five consecutive years (Figure 1). Unfortunately, we are afraid that there will be a dip next year, since the direct link to our home page at PubMed has been insufficient for almost a year. That has now been corrected in parallel with the connection of our journal to PubMed Central. This is an instrument for everyone to have free electronic access to all articles, in the case of UJMS from 1973 and onwards. Hopefully, this will mean that our figure for ‘total cites per year’ will increase from this year's 259, which was almost identical to that of last year (262). The figures of this year's Journal Citation Report reveal that our ‘Immediacy index’ (a measure of how often an article is cited during the publication year) has increased (from 0.067 to 0.083) and that figures for ‘Cited half-life’ (the median age of an UJMS article cited in 2009) and ‘Citing half-life’ (the median age of the items that UJMS cited in its 2009 articles) are well below 10 years.

**Figure 2.**
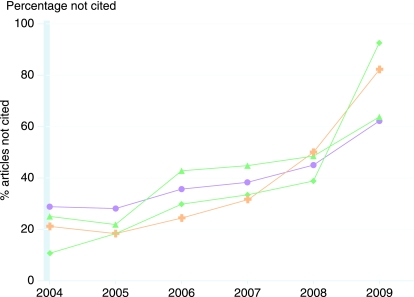
A graph from SCOPUS journal analyser showing the percentage of articles in that year that have never been cited as of 9 April 2010. Upsala Journal of Medical Sciences (diamonds), New England Journal of Medicine (triangles), The Lancet (circles), and Acta Dermato-Venereologica (crosses).

As mentioned above, an alternative database for information on the performance of medical journals is that of SCOPUS. Their SJR and SNIP figures are at present somewhat too unknown and vague to comment upon, but their figures for percentage of not cited papers in different journals are quite straight forward. In Figure 2 we have gathered data on percentages of articles not cited for UJMS and three other journals of interest—New England Journal of Medicine (the ‘dragon’), Acta Dermato-Venereologica (another Uppsala-based journal), and The Lancet. At least two major conclusions can be drawn: 1) Also in the ‘finest’ journals there are substantial numbers of papers that are not cited at all in the first 3–5 years after publication; and 2) the percentage of articles in our own journal that indeed have been cited is as high as for papers in the journals chosen for comparison. So the possibility of being cited seems as good in our journal as in any other more highly ranked journals. Perhaps one would dare to say that it is more important what is in the article than where it is published, at least when it comes to raw citation figures.

Another way of judging the success of a scientific journal and its articles was recently published by the Swedish Research Council in a publication named ‘Swedish production of highly cited scientific publications’ (*Vetenskapsrådets Lilla Rapportserie, 1: 2010*). Of special interest in that article is the methodological definition of what is the ‘most highly cited publications’ in the world. In order to belong to the 10% most cited papers an article has to be cited at least seven times during the publication year and the 2 years to come (self-citations not included). For the next level, the 1% most cited publications, the figure is 21 citations. It is obviously in the interest of the editor of a scientific journal to accept/gather as many articles of this kind as possible. The Research Council report has used figures of this kind to describe/compare the scientific success of different nations and universities. We are happy to find that in UJMS, amongst the publications of the last 9 years/volumes, there was one article (Nielsen et al., 2005, vol. 110, pp. 179–183) that had nine citations. Interestingly, that paper deals with a subject of great interest in Sweden these days—low-carbohydrate diet in type 2 diabetes. This field of research has been claimed to lack scientific contributions of high quality (*Mat vid diabetes—SBU rapport: 2010*). It is good to see that at least our journal has got one such article. There might be more to come. Thus, in our volume of 2008, with half a year more to go, one article has already got six citations. Hopefully, then another article from our journal will be classified as a ‘highly cited publication’ (data from SCOPUS).

Some final comments will deal with the experience from the collaboration with our new publisher, Informa Healthcare, and the use of the ScholarOne Manuscript Central. All in all it has been a pleasant and smooth one, with the insufficiency of the PubMed link as the only complication. As with other journals introducing the manuscript central there has been a marked increase of the manuscript inflow. At present we handle some 150 papers on an annual basis. This means that more than two-thirds of them have to be rejected, since we cannot lodge more than about 40 manuscripts per volume. Still, we recommend it to our colleagues in Uppsala, juniors and seniors, to submit their papers to UJMS. In last year's four issues less than 40% of the contributions were from Uppsala or had Uppsala collaborators. We think that this figure could be increased to a certain extent. We can offer short handling times (last year 26 days) with early online publication of accepted papers and also open-access facilities, which by the way will become mandatory for recipients of VR and EU grants in the near future. In doing so we should be able to have in hand at our premises a highly reputed medical journal that can serve as a quick and convenient publisher of acknowledgeable science.

